# Progress in the Diagnosis of Invasive Fungal Disease in Children

**DOI:** 10.1007/s12281-017-0274-9

**Published:** 2017-05-02

**Authors:** Adilia Warris, Thomas Lehrnbecher

**Affiliations:** 1 Aberdeen Fungal Group, MRC Centre for Medical Mycology, Institute of Medical Sciences, University of Aberdeen, Foresterhill, Aberdeen, AB25 2ZD UK; 2 Division of Paediatric Haematology and Oncology, Hospital for Children and Adolescents, Johann Wolfgang Goethe-University, Frankfurt, Germany

**Keywords:** Invasive fungal disease, Diagnostics, Paediatric patients, Imaging, Galactomannan, PCR

## Abstract

**Purpose of Review:**

This review summarizes the fungal diagnostic measures currently available for use in paediatric patients at high risk for developing invasive fungal disease (IFD) and those suspected of having an IFD. The clinical utility of each test is described based on reported performances of individual tests in specific paediatric populations.

**Recent Findings:**

Available studies in the paediatric population are scarce and are characterized by a huge heterogeneity in underlying diseases (e.g. different risk for IFD), different study objectives and management strategies (screening versus diagnostic) used.

**Summary:**

A final valuation of paediatric studies on fungal diagnostic tools is limited. While the galactomannan and fungal PCR assays are useful to exclude the presence of IFD, it is unclear if mannan, mannan antibodies and β-D-glucan are of benefit due to a lack of studies or validation of the cut-off, respectively. Well-designed multicentre paediatric studies are urgently needed to improve the outcome of IFD.

## Introduction

A timely diagnosis of invasive fungal disease (IFD) in children is a real challenge due to difficulties encountered in obtaining enough sample volumes, the need for anaesthesia to perform certain diagnostic procedures, and sparse clinical data with respect to the usefulness of fungal biomarkers and molecular detection methods. Culture and/or histopathological evidence of tissue invasion by the fungus is the gold standard to diagnose an IFD and is classified as proven disease [1]. Fungal biomarkers (including galactomannan, mannan and β-D-glucan) and fungal species-specific or pan-fungal PCR have been developed as tools both to enhance an early diagnosis of IFD as well as to increase the likelihood of the presence of an IFD. Several PCR based methods for the detection of resistance profiles including mutations conferring resistance to specific antifungals (e.g. azole resistance in *A. fumigatus*, echinocandin resistance in *Candida* spp.) are available as well. In addition, imaging modalities, in particular in the diagnosis for invasive pulmonary aspergillosis and to assess dissemination of a specific IFD, play an important role in the diagnostic process of a suspected IFD. It has been recognized that the performance and usefulness of both the more conventional as the newer fungal diagnostic tools in paediatric patients may differ from those in adults. This has led to 2 paediatric specific European guidelines for the management of IFD [2••, 3••]. At present, the EORTC-MSG definitions for the diagnosis of IFD undergo a second revision in which paediatric specific diagnostic recommendations will be included. The present review will focus mainly on paediatric specific studies in which the performance of a specific fungal diagnostic test has been evaluated.

## Fungal Culture & Histopathology

The sensitivity of blood cultures to detect candidaemia ranges between 21 and 71% as reported in autopsy studies [4] and is believed to be lower for neonates and infants due to sample volume issues. The ESCMID/EFISG guideline recommends to take 3 blood samples with a minimum volume of 2 ml each in children with a body weight < 2 kg and 6 ml each if between 2 and 12 kg to ensure a 50–75% sensitivity to detect *Candida* [5]. It may be obvious that these recommendations are impossible to be followed for (premature) neonates. Blood cultures are further limited by slow turn-around time and will not be positive in deep seated *Candida* infections including hepatosplenic candidiasis. *Aspergillus* species are rarely cultured from blood samples [6], and diagnostic accuracy of histopathological examination is below 80% [7]. Although most moulds are hardly recovered from blood cultures, positive blood cultures are more often obtained in IFD caused by *Scedosporium* spp., *Fusarium* spp. and *Acremonium* spp. (among others) due to the formation of spores during invasive growth. Mycological culture of BAL fluid in case of pulmonary mycoses has a low sensitivity of between 34% and 50% [8, 9]. Although histopathological examination will enhance differentiation between classes of moulds based on characteristic morphologies (e.g. *Aspergillus* species versus Mucorales) during invasive tissue growth, culture and/or molecular detection methods are needed to identify the causative fungus [8]. Often concerns are raised about the feasibility and safety of performing invasive diagnostic procedures in children and the added diagnostic value. It has been demonstrated though, that both bronchoscopy and lavage as well as CT-guided transthoracic biopsies can be performed safely in children and leads to more accurate and causative diagnosis of invasive fungal disease [10, 11]. Specific fungal agars to detect individual *Candida* species (chromogenic agars) or to detect azole-resistant *Aspergillus* species (4-wells agar plates, VIPcheck™) [12] will enhance timely identification and targeted antifungal therapy.

## Fungal Biomarkers

### Galactomannan

Galactomannan (GM) is a carbohydrate constituent of the *Aspergillus* cell wall that is released by all *Aspergillus* spp. during growth. This polysaccharide can be detected by a commercially available, FDA-approved enzyme immunoassay that incorporates the ß-1–5 galactofuranosyl specific EBA2 monoclonal antibody as a detector for GM (Platelia™ Aspergillus, Bio-Rad). Cross-reactivity has been observed for a number of other fungi (e.g., *Penicillium* spp., *Fusarium* spp., *Histoplasma capsulatum*, *Cryptococcus neoformans*), *Bifidobacterium* spp. and some batches of β-lactam antibiotics. On the other hand, non-angio-invasive aspergillosis as is seen in non-neutropenic immunocompromised patients and systemic mould-active antifungal prophylaxis have been associated with false-negative test results [13].

Initial studies clearly showed the usefulness of this test in the early detection of invasive aspergillosis (IA) with positive test results in neutropenic patients before clinical signs and symptoms, including CT-chest abnormalities, develop [13]. Galactomannan testing can be used both as a screening tool in patients considered at high risk for developing IA as well as a diagnostic tool in patients suspected of having developed IA [14, 15••]. It is included as a mycological criterion in the revised 2008 EORTC/MSG consensus diagnostic definitions [1], and GM testing is recommended by a number of current guidelines for early diagnosis and timely management of IA in both adult and paediatric patients [2••, 16].

A reasonable number of studies have assessed the usefulness of GM testing either as a screening tool or as diagnostic test for IA in defined paediatric populations in which the EORTC/MSG definitions were used to classify the probability of IFD (Table 1). Most of the studies were performed either to evaluate GM as a screening strategy in patients at high-risk for IFD according to their underlying malignancy, or as a diagnostic strategy in patients having a high clinical suspicion for IFD based on persistent febrile neutropenia not responding to broad spectrum antibiotics. Most of the study populations consisted of patients with underlying haematological malignancies and patients after haematopoietic stem cell transplantation (HSCT), and to a lesser extent, patients suffering from solid tumours, which, in general, have a lower a-priori risk for IFD than children with leukaemia and children undergoing HSCT. The studies included between 46 and 198 patients, assessing between 136 and 1865 samples, respectively. Only 3 of the studies in which GM testing was part of a screening strategy were performed in neutropenic children [17–19]. GM testing has not been validated in non-neutropenic patients and in the clinical setting the value of GM testing differs clearly between neutropenic and non-neutropenic patients [20].

**Table 1:** Overview of the studies in paediatric patients with underlying malignancies and/or paediatric haematopoietic stem cell recipients assessing the clinical utility of galactomannan (GM), β-D-glucan (BDG) and fungal PCR in either a screening strategy, diagnostic strategy or combination of both. Only those studies are shown in which the EORTC-MSG criteria [ref 1] are used and in which details about the cut-off (GM and β-D-glucan) and performance of the test are given

Marker	N (age)	IFD* prevalence	Schedule	Cut-off	Period	Strategy	sensitivity	specificity
GM								
Dinand [27] 2016	145 (0.3-18 yrs)	13.8%	n.a.	≥ 0.5, ≥1 sample	FN, clinical suspicion	Diagnostic	95%	80%
Gefen [19] 2015	46 (0.5-19 yrs)	8.7%^$^	1-2x/wk	≥ 0.5, ≥1 sample	neutropaenia	Screening	80%	66%
Choi [25] 2013	99 (0.3-18.7 yrs)	23.2%	n.a	≥ 0.5, ≥2 sample	FN, GvHD	Combination	91%	82%
Jha [26] 2013	78 (1.5-13 yrs)	2.0%	n.a.	≥ 0.5, ≥1 sample	fever	Diagnostic	84%	38%
Badiee [18] 2012	62 (1-14 yrs)	16.1%	2x/wk	≥ 0.5, ≥1 sample	neutropaenia	Screening	90%	92%
Fisher [17] 2012	198 (3.7-13.5 yrs)	0.5%	1-2x/wk	≥ 0.5, ≥1 sample	neutropaenia	Screening	0%	95%
Castagnola [24] 2010	119 (0.1-20 yrs)	3.6%	n.a	≥ 0.5, ≥2 sample ≥ 0.7, ≥1 sample	FN, GvHD, HSCT	Combination	32%	98%
Armenian [23] 2009	68 (0.4-22 yrs)	4.4%^$^	n.a	≥ 0.5, ≥2 sample	FN, GvHD, neutropenia	Combination	100%	98%
Hayden [22] 2008	56 (0.3-18 yrs)	30.4%	1x/wk	≥ 0.5, ≥1 sample	neutropenia, immunosupp	Screening	65%	87%
Steinbach [21] 2007	64 (0.8-19.5 yrs)	1.6%	2x/wk	≥ 0.5, ≥1 sample	neutropenia & GVHD	Screening	0%	87%
BDG								
Koltze [41] 2015	34 (0-16 yrs)	17.6%	1x/wk	> 80 pg/ml	Post-HSCT 100 days	Screening	90%	78%
Badiee [18] 2012	62 (1-14 yrs)	16.1%	2x/wk	> 80 pg/ml	neutropaenia	Screening	50%	46%
PCR								
Reinwald [56] 2014	95 (0.5-20.5 yrs)	0%^£^	1x/wk	n.a.	FN	Diagnostic	34%	78%
Badiee [18] 2012	62 (1-14 yrs)	16.1%	2x/wk	n.a.	neutropaenia	Screening	80%	96%
Mandhaniya [55] 2012	29 (1.5-15 yrs)	3.4%	n.a.	n.a.	Clinical suspicion IFD	Diagnostic	0	36%
Landlinger [54] 2010	125 (not given)	6.7%	n.a.	n.a.	Clinical suspicion IFD	Diagnostic	96%	77%
Hummel [53] 2009	71 (0-20 yrs)	7.0%	n.a.	n.a.	Clinical suspicion IFD	Diagnostic	80%	81%
Cesaro [52] 2008	62 (1.3-18 yrs)	12.9%	2x/wk	n.a.	FN, GvHD, clinical suspicion IFD	Diagnostic	88%	37%
Armenian [23] 2009	17 (0.4-22 yrs)	4.4%^$^	1x/wk	n.a.	Neutropaenia, GvHD	Screening	11%	79%
El-Mahallawy [51] 2006	91 (2-18 yrs)	30.8%	n.a.	n.a.	FN	Diagnostic	75%	92%

*proven/probable Invasive Fungal Disease

^$^those studies included also possible IFD

^£^possible IA was used as endpoint

FN, febrile neutropenia

GvHD, graft versus host disease

HSCT, haematopoietic stem cell transplant

The comparison of the studies is limited by the heterogeneity, such as by different definitions of test positivity (e.g, per sample or per 2 consecutive samples), and by the heterogeneity of the cut-off (e.g., optical density of 0.5 or 0.7). Similarly, the number of patients with proven/probable IFD vary widely between the studies (median 5.5; range, 1–23) as well as the prevalence of IFD in the various centres. Due to the heterogeneity in study design and study population, it is not surprising that there is a wide range of reported sensitivity and specificity. Combining the study results from the 5 studies in which GM was used as a screening tool only [17–19, 21, 22], sensitivity and specificity ranges from 0 to 90% (median 65%) and 66 to 95% (median 87%), respectively. Adding the 3 studies in which GM was used both as a screening and diagnostic measure [23–25], sensitivity and specificity ranges from 0 to 100% (median 72.5%) and 66 to 98% (median 89.5%), respectively. Only 2 paediatric studies did use serum GM as a diagnostic tool in children with prolonged febrile neutropenia showing a comparable sensitivity of 84% and 95%, but a clearly different specificity of 38% and 80% [26, 27]. These data favourably compare with the results given in a recent meta-analysis for GM testing in adults [sensitivity 0.82 (95% CI 0.73 to 0.90) and specificity 0.81 (95% CI 0.72 to 0.90)] [15••].

A limited number of studies have been performed on the evaluation of GM in other body fluids, such as broncho-alveolar lavage (BAL) fluid or cerebrospinal fluid (CSF). Two retrospective studies in immunocompromised children assessed the value of GM testing in BAL fluid for the diagnosis of invasive pulmonary aspergillosis (IPA) [10, 28]. Using a cut-off index of ≥0.5, a sensitivity of 82.4% and 78% was reported, with a specificity of 87.5% and 84%. These results do support the use of GM testing in BAL fluid if IPA is suspected based on clinical signs and symptoms and are comparable to those reported in adult high-risk haematology patients [29].

The utility of GM testing in the diagnosis of CNS aspergillosis in children is supported only by small retrospective case reports and case series [30, 31]. In one study, GM levels in the CSF in 5 patients with probable CNS aspergillosis were significantly higher than those of 16 control patients indicating the potential diagnostic value of GM in CSF [30].

In addition to its diagnostic use, serial serum GM assessments can used to monitor the effectiveness of antifungal therapy [32, 33]. Although these studies did not include paediatric patients, the possibility to identify patients failing antifungal therapy early during the course of IA will facilitate prompt interventions (e.g. optimizing dosages, switch to different antifungals) and optimize outcomes. Recently, Huurneman et al. aimed to develop a pharmacokinetic-pharmacodynamic model linking the PK of voriconazole in 12 paediatric patients suffering from IA with GM measurements. Such an approach does take into account that the voriconazole plasma level associated with a decreasing GM in an individual patient is predictive of a favourable outcome, and not the voriconazole level per se [34•].

### β-D-Glucan

Whereas GM has the limitation of being able to detect only IA, β-D-glucan (BDG) as a cell wall component of many pathogenic fungi can be detected in invasive infections due to *Aspergillus* spp., *Candida* spp., *Pneumocystis jirovecii*, *Fusarium* spp., *Trichosporon* spp. and *Saccharomyces* spp., whereas it is absent in mucormycosis. Similar to GM, BDG is included as mycological criterion in the revised definitions of IFD from the EORTC/MSG consensus group [1]. Studies in adults suggest that monitoring of BDG might be a useful method to exclude IFD in clinical environment with low to moderate prevalence of IFD. Many potential sources for contamination have been demonstrated and may lead to false-positive results [35••]. A meta-analysis showed a pooled sensitivity and specificity of BDG of 76.8% (95% CI, 67.1%–84.3%) and 85.3% (95% CI, 79.6%–89.7%), respectively, and importantly, no major differences in the sensitivity of BDG testing for the detection of invasive *Candida* or *Aspergillus* infections were observed [36].

The first studies in children haves shown that mean BDG levels are higher in immunocompetent uninfected children than adults [37], that *Candida* colonization of the gut in paediatric cancer patients leads to increased serum BDG (82–141 pg/ml) [38], and that very high levels of BDG exists in neonates and children with proven IFD [39]. Goudjil et al. showed that the optimal BDG cut-off for the identification of neonates with invasive candidiasis was 125 pg/ml (and not 80 pg/ml as suggested for adults) resulting in a sensitivity and specificity of 84% and 75%, respectively [40]. Two paediatric studies assessing the value of serum BDG in the diagnosis of IFD (mainly IA) showed that this test is not a reliable efficient diagnostic tool in the paediatric patients with hematologic disorders (*n* = 62) and HSCT recipients (*n* = 34) due to a high number of false-positive and false-negative results when 80 pg/ml was used as the cut-off (Table 1) [18, 41•]. Due to the paucity of data on BDG in children and the need to validate a paediatric specific cut-off, current guidelines recommend that BDG should not be used to guide paediatric clinical decision making [2••].

### Mannan and Anti-Mannan Antibodies

Mannan, a *Candida* cell wall component, and anti-mannan antibodies, have been used as a target for serological tests and are specific biomarkers for the detection of invasive candidiasis. Sensitivity is species-dependent and lower for *C. parapsilosis* and *C. krusei* (4–50%) than for *C. albicans* and *C. tropicalis* [42, 43], which needs to be taken into account when used in neonatal and paediatric populations. Mannan antigen detection alone shows an excellent specificity (97.5%) but is offset by an unacceptable low sensitivity (58.9%). Combination of mannan antigen and anti-mannan antibodies increases the sensitivity to 89.3% but will be at the cost of lowering the specificity to 63% [44]. Paediatric specific data is not available and only 1 smaller study included a restricted number of children [45]. Interestingly, the influence of *Candida* colonization in children with cancer on the performance of the mannan antigen assay has been studied and showed not to give rise to significant levels of mannan in the circulation [38]. Mannan antigen testing in serum and CSF may be of value to diagnose hepatosplenic candidiasis (blood cultures are rarely positive) and *Candida* meningitis, respectively [46, 47].

## Molecular Fungal Tools

The lack of standardization and absence of validated commercial systems of PCR-based methods for the detection of fungal pathogens resulted in the exclusion of PCR testing in the revised 2008 EORTC/MSG diagnostic criteria [1]. However, collaborative efforts (Fungal PCR Initiative, FPCRI) have led to the provision and widespread clinical evaluation of optimal standardized and validated protocols of the *Aspergillus* PCR and is proposed to be included in the revised EORTC/MSG diagnostic criteria [48]. Several commercial *Aspergillus* PCR assays are available which will provide a standardized approach if combined with the FPCRI recommendations. Blood and BAL fluid samples are most commonly tested by PCR for the purpose of screening high risk patients and diagnosing IPA, respectively. The test characteristics of the *Aspergillus* PCR showing a greater specificity for BAL fluid while a significantly greater sensitivity was demonstrated for blood [48]. Sensitivity and specificity of *Aspergillus* PCR in blood samples are 81% and 79%, respectively, as shown in a recent meta-analysis [49••].

A total of 9 paediatric studies were recently reviewed in which a PCR-based assay was used in children at high-risk or suspected of IPA [50•]. An *Aspergillus* specific PCR used as a screening tool in haematology patients at high risk for IA was evaluated in 2 studies and demonstrated a high negative predictive value [18, 23]. The other 6 studies assessed the use of a PCR assay (4 *Aspergillus* specific, 2 pan-fungal) as a diagnostic tool in immunocompromised children suspected of having IPA and showed overall a wide range of sensitivities (34%–96%) and specificities (37%–96%) (Table 1) [51–56].

Comparable efforts have been made to improve the detection of *Candida* spp. in patients with invasive candidiasis. Pooled sensitivity and specificity for suspected invasive candidiasis are 95% and 92%, respectively [57]. Several studies have shown that PCR results preceded positive cultures significantly, led to earlier initiation of antifungal therapy (median of 3 days) and showed to have prognostic value [4]. Scarce data is supporting the clinical utility of *Candida* PCR assays for the early and improved detection and identification of invasive candidiasis in neonates and children [58, 59]. Two commercial *Candida* PCR assays are currently available. One is a multiplex PCR assay detecting 19 bacteria and 6 fungi (SeptiFast) which showed a sensitivity of 61% and a specificity of 99% for the detection of candidaemia in adult patients with sepsis [60]. In a large paediatric study using SeptiFast to diagnosis septicaemia, the rate of positive results was significantly higher by this PCR assay (14.6%) compared to culture (10.3%), with a specific higher rate of detection of *Candida* spp. [61]. The recently FDA-approved T2 detection methodology using magnetic resonance to detect *Candida* colony-forming units is promising for the detection of invasive candidiasis in adults [62•]. A clinical study (NCT02220790) called “BIOmarkers in Paediatric Invasive Candidiasis” (BIOPIC) will prospectively enrol 500 children at high risk for developing invasive candidiasis and test 4 currently approved molecular assays for the detection of *Candida* spp. including the T2Candida platform.

Several PCR based methods for the detection of fungal pathogens including mutations conferring resistance to specific antifungals (e.g. echinocandin resistance in *Candida* species and azole resistance in *Aspergillus fumigatus*) are also currently available and will further enhance early targeted antifungal therapy [63, 64].

A potential drawback of PCR testing in the paediatric population is the amount of specimen needed to perform valid testing (about 2 ml), which is markedly more material than that needed for the GM (600 ul, including serial retesting), BDG (about 200 ul, including serial retesting), and the lateral flow device (LFD) (100 ul) tests.

## Imaging Modalities

Chest computed tomography (CT) plays an important role in the early diagnosis of invasive mould infections, in particular invasive aspergillosis, as the vast majority of those infections are localized in the lungs. Characteristic CT findings for IA including particular nodules with halo sign, air crescent sign and cavitation have been described in adults and are included as clinical criterion in the revised EORTC/MSG definitions [1, 65]. Those findings are however not restricted to IA and can also be caused by other moulds. The so-called reversed halo sign has been described as an early sign of invasive mucormycosis and may assist differentiation between those 2 groups of moulds exhibiting different antifungal susceptibilities [66]. In children, those typical signs are often not observed and radiographic findings are more unspecific (Table 2) [67–69]. The appearance of new abnormalities on the CT-chest during febrile neutropenia not responding to broad spectrum antibiotics should therefore be considered as possible IFD in children.

**Table 2:** The spectrum of radiographic abnormalities in paediatric patients with probable and proven invasive aspergillosis

	Burgos 2008 [69] 9.9 yrs. (0–18 yrs) 12% PID 88% cancer	Thomas 2003 [68] 5 yrs. (7 mo – 18 yrs) 50% PID 50% haem-onc		Taccone 1993 [67] 11 yrs. (7–18) 100% haem-onc
	CT- chest (125)	X-thorax (18)	CT-chest (8)	CT-chest (14)
nodules	65 (52%)	4 (22%)	4 (50%)	8 (57%)
halo sign	12 (10%)	-	-	2 (14%)
air-crescent sign	3 (2.5%)	-	-	4 (29%)
infiltrates	39 (31%)	14 (78%)	2 (25%)	8 (57%)
cavities	27 (21%)	-	-	3 (21%)
other	42 (34%)	5 (28%)	-	-

PID, primary immunodeficiency; haem-onc, haemato-oncology

Screening patients with probable or proven invasive pulmonary aspergillosis by magnetic resonance imaging (MRI) of the brain is recommended even in the absence of neurological signs and symptoms [70, 71•]. Dissemination of IPA to the CNS has been reported to be as high as 14% and clinical signs and symptoms only developed late in the course of the disease [72, 73]. Although existing guidelines do not recommend screening of extra-pulmonary sites in the absence of localized signs or symptoms, early detection of cerebral localisations of IA, followed by targeted treatment regarding the choice and dose of an antifungal with sufficient CNS penetration, including surgery in selected patients, will improve the dismal outcome of CNS aspergillosis.

Imaging modalities are crucial in the diagnosis of hepatosplenic candidiasis (= chronic disseminated candidiasis), a distinct phenotype of deep seated candidiasis localized mainly in the spleen and liver, affecting neutropenic patients with children being specifically at risk for this form of invasive fungal disease [74, 75]. MRI appears to be superior to both CT and ultrasound but due to the lack of an inflammatory response during neutropenia initial imaging results are often negative. Repeated imaging is required after neutrophil recovery to improve the diagnostic accuracy [76•].

## Future Perspectives

Lateral-flow device technology allows for rapid and bed-side testing with minimal technical expertise. For the diagnosis of IA, an immunochromatographic LFD using a monoclonal antibody (JF5) specific for a glycoprotein antigen release during active growth of *A. fumigatus* has been developed [77]. Preclinical data has shown that the test is highly specific, reacting with antigens from various *Aspergillus* species, but not with antigens from a large number of clinically important fungi including *Candida* spp., *Fusarium solani*, *Rhizopus oryzae* and *Cryptococcus neoformans*. Clinical experience with this new LFD is limited and only 2 studies in adults have evaluated the performance of this LFD on serum samples compared to real-time PCR and GM enzyme immunoassay [78, 79]. Comparable specificity (98% vs 95.5% and 91.5% for PCR and GM, respectively) and sensitivity (81.8% vs 95.5% and 77.3% for PCR and GM, respectively) were observed. In combination with PCR, a 100% sensitivity and specificity could be reached to differentiate proven/probable-IA from no-IA [79].

A high diagnostic potential of the LFD in BAL samples compared to PCR, GM, BDG and culture, in differentiating patients with proven and probable IA (17 patients) versus no IA (44 patients), was shown in adults [9]. Sensitivity of the LFD was slightly higher compared to PCR and GM (80% vs. 70%), while the specificity was comparable (between 95% and 100%). Combining either GM and PCR or GM and LFD resulted in an increased sensitivity (100% and 94%, respectively). Johnson et al. compared the diagnostic accuracy of the LFD with those of a real-time PCR (qPCR) and GM in BAL from patients with proven IA (*n* = 8) versus no IA (*n* = 15). The LFD showed a higher sensitivity and specificity when compared to GM (100% vs 87.5% and 80% vs 66.7%, respectively) and comparable sensitivities and specificities when compared to the qPCR used. Combined use of the LFD and qPCR did not improve the test characteristics any further [80]. Paediatric studies are still awaited.

In addition to searching for more sensitive and specific fungal biomarkers predicting IFD, identification of host response biomarkers may contribute to an early diagnosis of IFD. Discovery of IPA specific host response biomarkers have recently been described in a cohort of leukaemic adult patients receiving chemotherapy. The authors showed that by intergration of the host biomarkers with GM testing, the detection of probably IPA could be improved [81•].

Although the chest CT scan has a prominent role in diagnosing IPA, a clear differentiation between classes of causative pathogens is impossible, neither as is the differentiation to the underlying disease. New imaging modalities using specific radiopharmaceuticals are a promising tool for the detection of IA. In the pathophysiology of *A. fumigatus* infections, iron plays an essential role. Iron transporter mechanisms employed by *A. fumigatus* involve iron binding siderophores. By exchancing the iron for the radionuclide Gallium 68 (^68^Ga), siderophores can be labelled and visualized by the use of Positron Emission Tomography (PET) [82, 83]. In pre-clinical studies it has been shown that the use of ^68^Ga-labelled siderophores are specific targeting *A. fumigatus* and uptake is significantly lower in other fungal species and almost zero in other micro-organisms or cancer cells [84•]. Another approach is the use of *A. fumigatus-*specific antibodies labelled with a radionuclide to be visualized by PET/MR (magnetic resonance) imaging. By conjugating the *A. fumigatus*-specific monoclonal antibody JF5 with the chelator DOTA and then labelling with Copper 64 (^64^Cu), Rolle et al. were able to specifically detect IPA. In addition, by using this newly developed PET tracer, IPA could be distinguished from bacterial lung infections and nonspecific lung inflammation [85]. Of note, the same monoclonal antibody (JF5) was used as is present in the LFD.

## Conclusions

The clinical assessment of the various fungal diagnostic tools currently available are lacking behind in the paediatric population. Due to the fact that there are clear differences in underlying conditions and in terms of the epidemiology of IFD, the performances and clinical utility of the various tests used in the diagnosis of IFD may differ from those observed in adults [86]. In addition, the feasibility of performing invasive diagnostics and obtaining large enough sample volumes is more challenging in neonates and children. The paediatric studies performed to date with GM and fungal PCR are supportive of its use to rule out IFD due to their high negative predictive values as also seen in adult patients. For the β-D-glucan assay, a paediatric specific cut-off needs to be validated before this assay can be of clinical use in neonates and children. There are no studies assessing the value of mannan and mannan antibodies in the diagnosis of invasive candidiasis. Due to the central role the performance of chest CT scans has in the diagnosis of IPA in neutropenic patients, a paediatric study is urgently needed to confirm if the characteristic ‘halo sign’ and ‘air-crescent sign’ is indeed age-specific. Individual centres need to decide if the available fungal diagnostic tests will be employed in a screening strategy to rule out IFD (and thereby preventing overuse of antifungals) or a diagnostic test to rule in disease (targeted therapy). Positive arguments can be found for each strategy and depend on the underlying risk of IFD, prevalence and pre-test probability of disease and use of antifungal prophylaxis.

Overall, a final valuation of paediatric studies is clearly limited by several factors such as 1) small and inhomogeneous patient populations (e.g., haematological versus non-haematological malignancies; neutropenic versus non-neutropenic patients differing in the risk for IFD), 2) different study objectives (e.g., screening of patients without signs and symptoms of IFD versus diagnostic work-up of patients with suspected fungal infections), 3) different, often non-comparable PCR methods and 4) the evaluation of different samples such as blood, BAL fluid or CSF.

A multicentre trial to assess the use of non-culture based fungal diagnostic tools in which the shortcomings of the studies to date are overcome, is of utmost relevance to validate its use in the paediatric population. This trial needs to enrol a homogenous patient population at high risk for IFD, and for the analysis one needs ideally patients in whom IFD will be proven or ruled out during the study period. This trial will require a multicenter setting, but institutions have to be comparable regarding standardized diagnostic algorithms and procedures. In addition, optimal management strategies utilizing multiple diagnostic tools to increase performance and different approaches (e.g. screening versus diagnostic) to enable justified use of antifungals, reducing toxicity and costs. A recently initiated first paediatric trial is underway to assess the value of β-D-glucan, *Candida* T2 assay, mannan and mannan antibodies for the diagnosis of invasive candidiasis in paediatric patients, led by the International Paediatric Fungal Network (www.ipfn.org).
